# Q&A: Methods for estimating genetic gain in sub‐Saharan Africa and achieving improved gains

**DOI:** 10.1002/tpg2.20471

**Published:** 2024-06-26

**Authors:** Ibnou Dieng, Brian Gardunia, Giovanny Covarrubias‐Pazaran, Dorcus C. Gemenet, Bodo Trognitz, Sam Ofodile, Kayode Fowobaje, Solomon Ntukidem, Trushar Shah, Simon Imoro, Leena Tripathi, Hapson Mushoriwa, Ruth Mbabazi, Stella Salvo, John Derera

**Affiliations:** ^1^ International Institute of Tropical Agriculture (IITA) Ibadan Nigeria; ^2^ Bayer Crop Science Chesterfield Missouri USA; ^3^ Excellence in Breeding (EiB) c/o International Maize and Wheat improvement Center (CIMMYT) Texcoco Mexico; ^4^ EiB‐CIMMYT c/o ICRAF House United Nations Avenue Nairobi Kenya; ^5^ EiB‐CIMMYT c/o IITA Ibadan Nigeria; ^6^ IITA c/o International Livestock Research Institute (ILRI) Nairobi Kenya

## Abstract

Regular measurement of realized genetic gain allows plant breeders to assess and review the effectiveness of their strategies, allocate resources efficiently, and make informed decisions throughout the breeding process. Realized genetic gain estimation requires separating genetic trends from nongenetic trends using the linear mixed model (LMM) on historical multi‐environment trial data. The LMM, accounting for the year effect, experimental designs, and heterogeneous residual variances, estimates best linear unbiased estimators of genotypes and regresses them on their years of origin. An illustrative example of estimating realized genetic gain was provided by analyzing historical data on fresh cassava (*Manihot esculenta* Crantz) yield in West Africa (https://github.com/Biometrics‐IITA/Estimating‐Realized‐Genetic‐Gain). This approach can serve as a model applicable to other crops and regions. Modernization of breeding programs is necessary to maximize the rate of genetic gain. This can be achieved by adopting genomics to enable faster breeding, accurate selection, and improved traits through genomic selection and gene editing. Tracking operational costs, establishing robust, digitalized data management and analytics systems, and developing effective varietal selection processes based on customer insights are also crucial for success. Capacity building and collaboration of breeding programs and institutions also play a significant role in accelerating genetic gains.

## Core Ideas

- Annual genetic gain assessment drives breeding progress and efficiency.
- Realized genetic gain is preferably estimated using historical data with at least two long‐term checks.
- Realized genetic gain is estimated using the genotypes best linear unbiased estimators from the linear mixed model, regressed on their years of origin.
- State‐of‐the‐art breeding techniques, especially genomic selection, improve genetic gains.

**Abbreviations**
BLUE: best linear unbiased estimators
BLUP: best linear unbiased predictions
BMGF: Bill & Melinda Gates Foundation
EBS: Enterprise Breeding System
EBV: estimated breeding value
EiB: Excellence in Breeding
GS: genomic selection
LMM: linear mixed model
MET: multi‐environment trials
NARES: National Agricultural Research and Extension System
RGA: rapid generation advancement
SSA: sub‐Saharan Africa
TGE: transgenerational genome editing
TPE: target population of environments
TPP: Target Product Profile
Tricot: triadic comparison of technology

## 1 INTRODUCTION

The human population size is anticipated to grow, boosting food demand. The world's population will increase from 8.05 billion in 2023 to 9.71 billion by 2050, of which 6.7 billion will live in urban areas (United Nations, Department of Economic and Social Affairs, Population Division, 2022). More than 60% of the increase is expected in Africa, particularly in sub‐Saharan Africa (SSA), whose population is forecasted to grow 2% a year, an increase of 915 million people by 2050 (United Nations, Department of Economic and Social Affairs, Population Division, 2022). SSA will contribute significantly to the rise in the global food demand, especially for cereals and other staples like roots, tubers, bananas, and pulses. In this context, expanding the supply of plant‐based food products in the future is imperative to address the growing global demand for food. But SSA is a net importer of soybean [*Glycine max* (L.) Merr.] mainly for animal feed. Between 2013 and 2016, the region spent $4.4 billion on soybean imports, and this trend is expected to continue (Engelbrecht et al., 2020). Overall, a trade deficit in major food items in SSA is expected to increase from $9 billion in 2020–2022 to $24 billion by 2032 (OECD/FAO, 2023). To compound this need, by 2050, nearly 58% of SSA people are forecasted to live in urban settings (United Nations, Department of Economic and Social Affairs, Population Division, 2022). As more people shift into urban areas, it is anticipated that the loss of agricultural labor in rural areas could lead to decreased production and will require changes in agriculture to maintain productivity. In addition, urbanization converts agricultural land into urban infrastructure, reducing the land available for food production.

Also, future agricultural supply needs to respond to increasing per capita incomes and shifting consumer preferences. Individuals with higher incomes tend to consume more meat and dairy products (Godfray et al., 2018). This leads to a higher demand for feed crops such as cassava (*Manihot esculenta* Crantz), maize (*Zea mays* L.), yam (*Dioscorea* spp.), soybean, and others to meet the demand for animal‐based products (Delgado, 2003). Plant protein–based diets, such as those based on soybean or cowpea [*Vigna unguiculata* (L.) Walp.], can also replace meat‐based diets without compromising nutrition or food security (Eshel et al., 2019), and they can be more environmentally sustainable than meat‐based diets (Banach et al., 2022). In that context, national development strategies for some SSA countries classify crops like soybean as a strategic national priority crop.

Climate change is another significant factor predicted to affect the availability and demand of food from crops (Arora, 2019). Extreme weather occurrences and changes in weather patterns can result in crop failure and food shortages. Banana (*Musa* spp.) production is particularly threatened by drought, which can cause substantial yield losses (Ravi et al., 2013; Van Asten et al., 2011).

As the world population continues to grow and climate‐related disasters such as droughts and floods become more frequent, there is a pressing demand to develop resilient crop varieties capable of thriving under these challenging conditions. Plant breeding offers solutions in tackling food security concerns by tailoring crops to withstand the impacts of climate change. Therefore, effective monitoring of the success of plant breeding programs is essential. To gauge the efficacy of breeding efforts, plant breeders regularly assess the rates of genetic gain, denoting the improvement in performance attained through artificial selection (Xu et al., 2017). There are two methods for estimating genetic gain: expected and realized. Expected genetic gain quantifies the average change in a population's phenotype attributable to a specific breeding strategy (Rutkoski, 2019), while realized genetic gain captures the observed change in average population performance over time (Mackay et al., 2011; Piepho et al., 2014; Rutkoski, 2019). Realized genetic gain rate can further be measured in absolute or relative terms, offering insights into breeding strategy effectiveness and aiding in selecting individuals with superior traits (Seck et al., 2023).

Achieving enhanced genetic gains demands a multifaceted approach, necessitating the integration of cutting‐edge breeding practices, robust data management and analytics systems, an excellent varietal selection process that considers customer feedback, and optimizing the breeding process operational costs. Additionally, continuous capacity building initiatives and collaborative partnerships with industry stakeholders are pivotal in driving success, particularly in developing and disseminating demand‐driven improved products. Central to this effort is the development of effective breeding materials coupled with the implementation of good agronomic practices. This article reviews methods for estimating realized genetic gain rates and outlines strategies for achieving enhanced gains.

## 2 WHAT CAN WE LEARN FROM GENETIC GAIN RATE ESTIMATION?

A major global societal challenge is eradicating global hunger—one of the sustainable development goals (UN General Assembly, 2015)—and feeding the future world population in the context of climate change and arable land loss. Plant breeding can contribute to address this with innovative approaches to produce better varieties to increase crop yield potential and global production. Therefore, it is of utmost importance to monitor breeding programs’ successes by estimating genetic trends. This will require the development of comprehensive monitoring and evaluation systems to track the progress and impact of breeding programs through a feedback loop mechanism (Covarrubias‐Pazaran et al., 2022; Tiwari et al., 2022). In this context, the Breeding Program Assessment Tool has been used to evaluate plant breeding programs to improve their efficiency and achieve higher genetic gain (The University of Queensland, 2023). Breeding programs can also be monitored by tracking the genetic gain achieved over time. For a normally distributed trait in a population with genetic basis, directional selection—the best individuals beyond a threshold are selected over generations—is imposed by a breeder to select progenitors of the next generation under the assumption that those individuals provide better genetic properties (Recker et al., 2014). If this procedure is applied over several years, the offspring populations are supposed to perform better than the parents. But we know that plant breeding is affected by the methods of crossing, evaluation, and selection; logistics; certain nuisance factors mainly related to the environment; and so on Periodically estimating genetic gain will assess whether the offspring superiority hypothesis holds. If not, breeding strategies and logistics adjustments would be necessary for increased efficiency. The regular measurement of realized genetic gain is then crucial for evaluating new breeding strategies. As an example, analysis of two different historical time periods has thrown light on how changes positively or negatively can impact genetic gains. Yadav et al. (2021) examined wheat (*Triticum aestivum* L.) breeding programs in India over two different time periods. They found that the genetic gain in yield was greater in the second time period characterized by modern breeding methods and improved germplasm, compared to the first time period. By quantifying the extent of genetic improvement achieved, the estimation assists in strategically allocating resources within breeding programs, directing investments toward strategies that promise the highest rates of genetic advancement. While measuring genetic gain provides a quantitative assessment of progress achieved with specific breeding strategies, it inherently reflects past achievements. Therefore, critically evaluating new breeding strategies is recommendable to prioritize and implement approaches that are more likely to produce significant genetic progress. For example, Bandillo et al. (2023) used 1500 soybean lines tested in replicated experiments over 7 years to compare genomic selection (GS) to phenotypic selection. The study found that GS was comparable to phenotypic selection while suggesting some recommended measures to prevent the loss of genetic variance when employing GS. However, comparing strategies comes at a cost, and this is where simulations can play an important role in the decision‐making process. Cost‐effective simulations can inform decisions in designing breeding strategies by assessing the impact of changes in crossing, evaluation, or selection procedures on genetic gain. Several studies have been conducted using that approach (Bakare et al., 2023; Lin et al., 2023; Wu et al., 2023).

**Core Ideas**

- Annual genetic gain assessment drives breeding progress and efficiency.
- Realized genetic gain is preferably estimated using historical data with at least two long‐term checks.
- Realized genetic gain is estimated using the genotypes best linear unbiased estimators from the linear mixed model, regressed on their years of origin.
- State‐of‐the‐art breeding techniques, especially genomic selection, improve genetic gains.

Since 2021, CGIAR has been engaged in annually estimating realized genetic gain rates for its breeding programs. After 3 years of estimation, several challenges and lessons have emerged, particularly within the context of SSA. Some historical data from specific programs have not yet been migrated to breeding informatics systems. This creates several challenges. First, data are dispersed across numerous spreadsheets, and compatibility issues between data formats or structures often complicate consolidation efforts during genetic gain estimation. Second, concerns regarding data quality, such as inconsistencies and errors inherent in spreadsheet data, also pose significant obstacles. Third, metadata that describes the trials is not always available, making it difficult to accurately filter eligible trials for the estimation. Furthermore, the genetic gain estimation method considers data collected from multiple locations every season/year, making it more suitable for annual crops. However, for perennial crops like banana, where multiple harvests are required before selection decisions can be made, a longer period must be considered for estimation.

## 3 WHAT DATA ARE REQUIRED FOR GENETIC GAIN RATE ESTIMATION?

**FIGURE 1:**
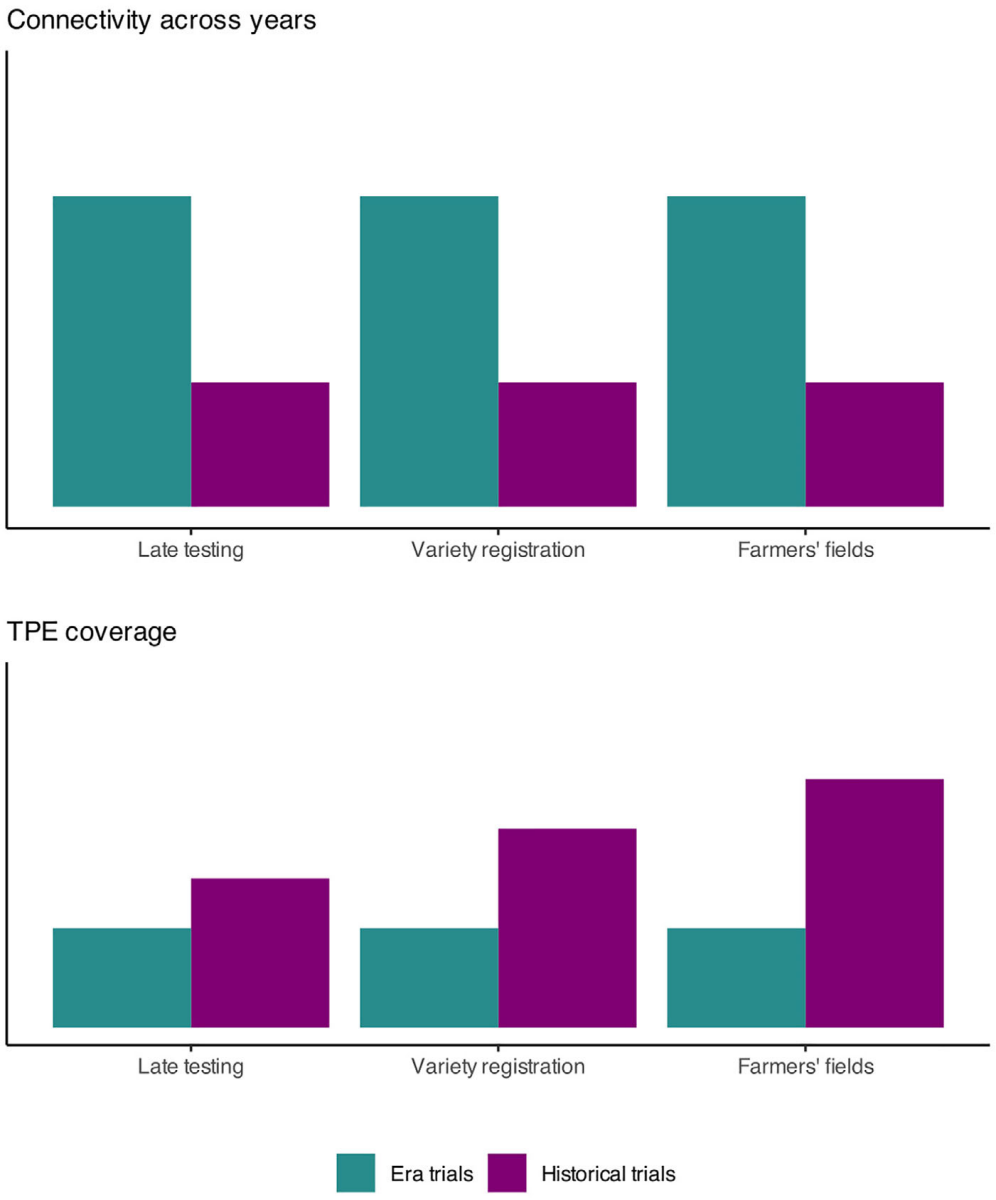
Schematic presentation comparing target population of environments (TPE) coverage and connectivity between historical and era trials at different breeding stages. Era trial information tends to maximize connectivity, whereas historical data depend on checks to have the same power. Historical data provide greater TPE coverage, while era trial information tends to provide less TPE coverage.

The rate of realized genetic gain can be estimated using historical‐trial data. These data include information on genetic material that has been evaluated in trials conducted across different locations over several years. These trials are carried out in collaboration with partners as part of the breeding program strategies to advance improved lines to the National Agricultural Research and Extension System (NARES) and the private sector. It can also be estimated by evaluating in the same experiment, with replicates, germplasm developed during a defined study period, commonly referred to as era trials. In these experiments, very few genotypes are tested in very few environments, limiting the scope of conclusions. Also, years of ongoing testing will favor newer genotypes that are more likely to be adapted to them (Rizzo et al., 2022). Connectivity, the number of lines in common between environments, and target population of environments (TPE) coverage are vital in estimating genetic gain. While era trials provide better connectivity, historical data are well suited for better TPE coverage. However, the effects of the environment are mostly estimated from the sample of connected checks, often small (Krause et al., 2023), and new breeding programs naturally do not have large historical datasets. Nevertheless, selection accuracy can be improved with the use of advanced statistical analysis methods that employ informative models for genotype‐by‐environment interaction and appropriately accommodate within‐trial error variation. Estimating realized genetic gain over a few years (2–10) can lead to inaccurate results (Krause et al., 2023; Rutkoski, 2019). For that, Krause et al. (2023) recommend using the linear mixed models (LMMs; Henderson et al., 1959) with various parameterizations of the G × L × Y, selecting the most appropriate model based on criteria such as the Akaike information criterion. In any case, the gains will only be achieved if the methods are applied with a suitable check strategy. CGIAR Excellence in Breeding (EiB) recommends including at least two stable long‐term check varieties to maintain connectivity across the years, a recommendation also made by Raymond et al. (2023). Additionally, it is recommended that varieties widely commercialized in the target market and one or two locally important varieties be used as checks. In this context, the use of historical data is the preferred option (Figure 1). Many studies have been carried out to estimate genetic trends by using historical data. Mackay et al. (2011) established standards and methods for this purpose. Laidig et al. (2017) utilized 32 years of data from official German trials to measure the yield progress of winter wheat varieties. More recently, several other studies have also been conducted using historical data (Khanna et al., 2022; Kumar et al., 2021; Menkir et al., 2022; Prasanna et al., 2022).

## 4 HOW CAN WE ESTIMATE REALIZED GENETIC GAIN?

Estimating genetic gain for a breeding program requires separating genetic trends due to breeding efforts from nongenetic trends due to agronomic advances and environmental changes (Piepho et al., 2014). This can be achieved by using the LMM on historical data from routine multi‐environment trials (MET) by (i) removing the year effect, that is, enhancement due to nongenetic reasons; (ii) estimating the improvement due to genetics; while (iii) accounting for the experimental designs, assuming heterogeneous residual variances of the environments, and modeling the residuals as a sum of measurement error and a spatially dependent random process, and so on.

Considering genotype as a random factor in an LMM, that is, estimating the best linear unbiased predictions (BLUP), when calculating realized genetic gain has been an approach considered by Woyann et al. (2019). Rutkoski (2019) ran stochastic simulations of 80 rice breeding programs over 28 years and compared different methods of realized genetic gain estimation. After evaluating factors such as performance, feasibility, and cost, the most effective approach was using estimated breeding values (EBVs), that is, considering the genotype as random and estimating the additive relationship matrix using pedigree records.

Krause et al. (2023) listed authors who adopted algorithmic modeling (Byrum et al., 2017) or a blend of algorithmic and linear modeling (Bornhofen et al., 2018; Brisson et al., 2010; Oury et al., 2012) to remove environmental factors and compute genetic gain rather than relying on LMM.

EiB recommends adopting the model proposed by Mackay et al. (2011) and Piepho et al. (2014) to calculate realized genetic gain in a breeding program where best linear unbiased estimators (BLUE) values of the genotypes are estimated in an LMM approach using residual maximum likelihood (Patterson & Thompson, 1971). The inherent genetic trends are then calculated from the regression of the BLUE values of the genotypes on the year of origin or release of the germplasm (Laidig et al., 2014; Mackay et al., 2011; Piepho et al., 2014). Here, the year of origin is the year in which the genotype was created or the first year it entered the testing program. Given that the varieties in historical or era trials may not always represent those currently cultivated, we followed Piepho et al. (2014) for the assessment of what they termed the “potential” genetic gain that can be realized if the latest available genotypes are grown each year.

Assessing genetic gain at various breeding stages provides detailed insights into breeding strategy performance. Early development stages typically involve the initial selection and evaluation of breeding lines in controlled environments such as research stations. This phase allows breeders to identify promising lines with desired traits and genetic potential. Late development stages entail further testing and validation of advanced breeding lines under more diverse and representative field conditions, more accurately evaluating adaptability and agronomic performance. Pre‐commercial and commercial stages involve large‐scale field trials across varied environments and practices to assess consistency and economic viability. Estimating genetic gain in farmers’ fields provides feedback on real‐world performance, yet it presents challenges due to environmental variability, including input use and management practices. Logistics aspects, particularly the quantitative measurement of variety performance at farmers’ fields, also pose significant challenges regarding methods and standards. While estimating genetic gain separately for each breeding stage is possible, prioritizing late‐stage testing, as CGIAR recommends, offers a standardized approach that effectively captures varieties’ adaptability and overall performance (Covarrubias‐Pazaran, 2020). Nevertheless, current research is ongoing at CGIAR to develop systems to measure the genetic gain made in farmers’ fields.

Standardizing the method of genetic gain estimation ensures uniformity and consistency in monitoring achievements across different programs, crops, and regions. As previously stated, determining genetic improvement involves identifying a genetic and a nongenetic trend in an LMM. Considering independent random genotype and year main effects would produce biased outcomes because shrinkage will lead to underestimation of genetic effects. Hence, treating genotypes and years as fixed effects can be considered to account for potential nonlinear trends in both genotype and year effects (Mackay et al., 2011).

The realized genetic gain estimation involves considering a three‐step analysis approach.

In the first step, individual trials can be analyzed to estimate heritability for each environment. The genotype is considered random in an LMM using the method of Cullis et al. (2006), which is suitable for analyzing trials with unbalanced datasets. For the example of an alpha‐lattice design, the LMM is of the form:

$$y_{ijk}=\mu +G_{i}+R_{k}+B_{jk}+e_{ijk}$$

where $y_{ijk}\;$ is the vector of phenotypic data of the 𝑖th genotype of the j th block nested into the k th replication; μ is the overall mean; $G_{i}$ is the effect of the 𝑖th genotype; $R_{k}$ is the effect of the k th replication; $B_{jk}$ is the effect of the j th block nested into the k th replication; and $e_{ijk}$ is the residual error with the assumptions $e_{ijk}∼N\left(0,\sigma_{e}^{2}\Sigma_{r}⊗\Sigma_{c}+\sigma^{2}I\right)$. $\Sigma_{r}$ is ther×r the spatial correlation matrix for the row, $\Sigma_{c}$ is the **c** **×** **c** spatial correlation matrix for the column; $\Sigma_{c}$ and $\Sigma_{r}\;$ are assumed to arise from autoregressive process of order 1, and $\sigma^{2}I$ is the measurement error (also known as nugget effect). All effects except the replication are considered random for estimating broad‐sense heritability using the method presented in Cullis et al. (2006).

$$H^{2}=1-\frac{V}{2\sigma_{g}^{2}}$$

where V is the pairwise predicted error variance of the BLUP of the genotypes and $\sigma_{g}^{2}$ is their variance.

A one‐ or two‐stage analysis can be performed in the second step after discarding all environments with low broad‐sense heritability. Specific exclusion criteria will depend on the trait under consideration. Based on simulations (not shown), EiB recommends excluding all environments with broad‐sense heritability less than 0.2. The standard method for analyzing MET data is a one‐stage analysis that involves a combined analysis of the plot data across trials (Gogel et al., 2018). A two‐stage analysis is an alternative approach, which requires estimating the mean genotype estimates (BLUE) with their corresponding weights, the diagonal of the inverse of the variance–covariance matrix of the prediction errors. However, with enough computing power, the one‐stage approach can be used for large and complex datasets, eliminating the need for the two‐stage approximation (Gogel et al., 2018).

The model has the following form in the case of a one‐stage (Piepho et al., 2014); we removed the design effects for simplicity:

$$\begin{array}{ccc} y_{ilt} & = & \mu +G_{i}+L_{l}+Y_{t}+{\left(\mathrm{LY}\right)}_{lt}+{\left(\mathrm{GL}\right)}_{il}+{\left(\mathrm{GY}\right)}_{it}\\ & & +\;{\left(\mathrm{GLY}\right)}_{ilt}+e_{ilt} \end{array}$$

where $y_{ilt}\;$ is the performance of the 𝑖th genotype in the 𝑙th location and 𝑡th year; 𝜇 is the overall mean; $G_{i}\;$ is the effect of the 𝑖th genotype; $L_{l}\;$ is the effect of the 𝑙th location; $Y_{t}\;$ is the effect of the 𝑡th year; ${\left(\mathrm{LY}\right)}_{lt}$ is the 𝑙th location × 𝑡th year interaction effect; ${\left(\mathrm{GL}\right)}_{il}$ is the 𝑖th genotype × 𝑙th location interaction effect; ${\left(\mathrm{GY}\right)}_{it}$ is the 𝑖th genotype × 𝑡th year interaction effect; ${\left(\mathrm{GLY}\right)}_{ilt}$ is the 𝑖th genotype × 𝑙th location × 𝑡th year interaction effect; and $e_{ilt}$ is the random residual error associated with observation $y_{ilt}$. We assumed heterogeneous residual error variances across environments (an environment is a location × year combination), that is, $e_{ilt}∼N\left(0,\;\sigma_{lt}^{2}\right)$. All effects except 𝜇, $G_{i}$, and $Y_{t}\;$ are assumed to be random. The main output in the second step of realized genetic gain estimation is the prediction of the genotype BLUE across locations and years, alongside their weights, and the diagonal of the inverse of the variance–covariance matrix of the prediction errors from the combined LMM.

In the final step, a weighted‐linear regression of the estimated performances (BLUE) of the genotypes on the year of origin is performed. Checks and germplasm from external sources must be excluded from the genotypes prior to the regression. We model $G_{i}$ as follows:

$$G_{i}=\alpha +\beta X_{i}+e_{i}$$

where α is the intercept, β is the fixed regression coefficient for genetic trend equivalent to the absolute rate of realized genetic gain per year in the unit of the trait, $X_{i}$ is the year of origin of the 𝑖th genotype, and $e_{i}\;$ a random residual, $e_{i}∼N\left(0,\;\sigma_{(G)i}^{2}\right)$. The percentage change in genetic gain due to genetic causes is estimated as a ratio of the regression slope (β) to the *y*‐intercept of the regression (α) plus the slope (β) multiplied by the year of first testing ($X_{0}$) or the mean years of the time interval, that is, $100\;\times (\frac{\beta }{\alpha +\beta X_{0}})$.

An illustrative example of estimating realized genetic gain is examined by analyzing historical data on fresh cassava yield in West Africa (https://github.com/Biometrics‐IITA/Estimating‐Realized‐Genetic‐Gain), providing a model applicable to other crops and regions.

Nongenetic trends can also be evaluated by modeling $Y_{t},\;$ the 𝑡th year main effect (Piepho et al., 2014) as follows:

$$Y_{t}=\delta +\zeta T_{t}+e_{t}$$

where δ is the intercept, ζ is the fixed regression coefficient for nongenetic trend, $T_{t}\;$ is the calendar year, and $e_{t}\;$ a random residual, $e_{t}∼N\left(0,\;\;\sigma_{(Y)t}^{2}\right)$. The nongenetic trends model can help assess changes over time in agronomy practice and environmental and climate factors.

Additionally, it is crucial to consider the impact of disease progression when evaluating genetic gain, as this factor can be associated with both long‐term genetic and nongenetic trends (Mackay et al., 2011; Piepho et al., 2014).

Breeding programs strategically prioritize traits of interest when assessing genetic gain, often giving precedence to productivity traits like yield. The traits to consider depend on the final objectives of the program. In addition to direct trait measurements, genetic gain estimation can also be enhanced through selection indices (Crevelari et al., 2019; Entringer et al., 2016), which allow for the simultaneous consideration of multiple important traits in breeding programs.

In essence, monitoring the successes of breeding pipelines is crucial to assess their efficacy. It can be achieved by estimating realized genetic gain using historical data. However, to drive further improvement, contemporary strategies need to be embraced. Integrating advanced technologies such as precision genetics, genomic tools, and advanced analytics into breeding pipelines—along with testing their efficacy—can empower breeders to make informed decisions and accelerate the desired genetic progress. The success of strategies to increase genetic gains first depends on a robust mechanism for germplasm development. Germplasm provides the raw material for breeding programs, enabling breeders to select for specific traits—whether it is disease resistance in soybean, vitamin content in sweet potato, or drought tolerance in maize. Integrating germplasm improvement (pre‐breeding) and new variety development while adopting cutting‐edge strategies alongside sustainable management practices creates a synergy that advances agricultural progress. The following section will discuss some of the modern breeding strategies.

## 5 WHAT STRATEGIES TO ACHIEVE INCREASED GENETIC GAINS?

The continuous advancement of plant breeding techniques and the utilization of diverse genetic resources are crucial for meeting future demands and ensuring resilient and productive crop‐based food systems (Salgotra & Chauhan, 2022). Technology and engineering will impact how crop‐based food production develops in the future. Adopting precision genetics, genomic tools, appropriate data management, and analytics methods to increase efficiencies and genetic gains is essential, although insufficient. In addition, breeding programs need equipment and well‐trained personnel to execute consistently, with high precision, the phenotyping, laboratory methods, seed management, and nursery activities required for a successful program.

Plant breeding programs are structured processes carried out by breeders and their technical support to maintain and advance populations of genetic recombinants from which superior varieties can be iteratively selected. A single breeding program consists of one or more breeding pipelines in which parental population improvement and selection of varieties is carried out to match the demand of specific market segments. The market segments are determined by the environments where the end‐product is grown, the requirements and preferences of the target clients, and the product traits that are important to farmers and end‐users during production and consumption. Products to be bred for a specific market segment are selected using a Target Product Profile (TPP), that is, the total of characteristics that have a genetic base and can be influenced through the breeding process. The TPP describes the “set of traits, the scale used to measure each trait, and the threshold score for each trait that is required in a new product to meet or exceed the needs of farmers, processors, and consumers in a crop market segment” (P. Coaldrake, personal communication, November 20, 2023).

In SSA, plant breeding programs encounter several constraints, such as slow delivery and adoption of improved crop varieties, resulting in low yields (Atlin et al., 2017). Uncertainties in funding availability and levels to sustain breeding efforts in research institutes (Eriksson et al., 2018), lack of access to markets, high costs, and shortage of plant breeders also pose challenges (Suza et al., 2023). To address these issues, a coordinated effort to modernize plant breeding programs across several countries in SSA, Asia, and Latin America is currently being implemented by CGIAR and its NARES partners. The initiative, known as the Accelerated Breeding Initiative, is composed of several strategies aimed at increasing the genetic gain rates of crops. These strategies include advanced breeding techniques, robust data management, customer insights, optimizing operational costs, capacity development, and collaborations.

### 5.1 Integrating state‐of‐the‐art breeding tools

#### 5.1.1 Genomic selection

GS can improve breeding pipelines by using advanced genotyping techniques to quickly analyze the deoxyribonucleic acid of individuals and identify genetic markers (Bernardo, 1994; Budhlakoti et al., 2022; Meuwissen et al., 2001; Robertsen et al., 2019; Wang et al., 2018). Using statistical models, GS links genotypic information with phenotypic data to establish relationships between genetic markers and the traits of interest. Accurate and extensive phenotyping is important for these models to estimate an individual's breeding value (EBV), which predicts the likelihood of passing desirable alleles to its offspring, improving the germplasm. GS enables breeders to make more accurate selection decisions, even at early stage (Beyene et al., 2021). By considering the underlying genomic information, breeders can improve the accuracy of predicting an individual's performance and potential contribution to future generations. GS can improve genetic gain by reducing cycle time and increasing accuracy and selection intensity (Larkin et al., 2019; Wartha & Lorenz, 2021), all fundamental in the breeder's equation. Genetic variance is also influenced by the training population optimization (Merrick et al., 2022). GS can help to overcome the difficulties of selecting traits, such as drought tolerance or yield under heat stress, which can be influenced by multiple genes (Zingaretti et al., 2020). Several programs have started implementing and mainstreaming GS into breeding pipelines of several crops important for SSA. Beyene et al. (2019) compared the effectiveness of tropical maize hybrids selected through phenotypic selection and GS, both in well‐watered and managed drought stress conditions. The authors found that GS and phenotypic selection produced similar results for grain yield and other traits, even under drought stress. But GS is more cost‐effective and can improve the efficiency of maize breeding. As tropical maize is unsuitable for temperate environments due to its sensitivity to longer photoperiods and cooler temperatures, Choquette et al. (2023) conducted a study using GS in an off‐season nursery to help accelerate the development of climate‐adapted varieties. Their results showed that this method can increase genetic gains for flowering time by over 50% compared to direct selection in summer seasons only, reducing the time required to change the population mean by one‐third to one‐half. Badu‐Apraku et al. (2019) conducted a study to determine the gains in grain yield and associated changes in an early‐maturing yellow biparental maize population following GS for improved grain yield, Striga resistance, and drought tolerance. The results showed an increase in yield under both Striga‐infested and optimal environments. However, GS did not improve yield under drought, and for it to continue progressing, it is essential to introduce new genes for resistance or tolerance to drought. Wolfe et al. (2017) outlined the perspectives of GS in cassava breeding. They found that accuracy for dry matter content and mosaic disease severity was sufficient for rapid‐cycling GS. Increasing the training population size was more important than selecting a type of prediction model. Gholami et al. (2021) discussed the progress made in implementing GS in the CGIAR. GS has been applied to crops such as cassava, maize, rice (*Oryza sativa* L.), wheat, and yam. The authors noted that other crops, including banana, forages, legumes, potato (*Solanum tuberosum* L.), and pulses, are developing and validating the necessary logistics and tools. Ultimately, the goal is to reduce the breeding cycle to 2–3 years.

#### 5.1.2 Genome‐editing

Genome‐editing technologies employing site‐directed nucleases such as meganucleases, zinc‐finger nucleases, transcription activator‐like effector nucleases, and clustered regularly interspaced short palindromic repeats/CRISPR‐associated protein (CRISPR/Cas) have emerged as powerful tools for manipulating plant genomes. Among these, CRISPR/Cas9 genome editing stands out as a revolutionary technique in plant biotechnology, enabling precise modifications in the plant genome (Chen et al., 2019; Tripathi et al., 2022). These modifications lead to crops with increased resistance to pests and disease, improved yield, and nutritional content. Gene editing can be used at both forward breeding and trait introgression. In forward breeding, gene editing can be used to introduce specific beneficial traits into the elite variety without necessarily involving genes from a different species. This could involve editing endogenous genes to enhance desirable traits such as disease resistance, yield, nutritional content, or environmental adaptability. Gene editing allows for precise changes to be made to the crop genome, potentially speeding up the breeding process compared to traditional breeding methods. Gene editing can also be used to facilitate trait introgression by precisely editing the genome of the recipient organism to incorporate the desired trait from another species, for example, wild type. This process may involve editing multiple genes to achieve the desired trait introgression, and it may require more complex genetic engineering techniques compared to forward breeding. An example could be transferring a gene from a wild plant species known for its resistance to a particular disease into a cultivated crop plant to confer disease resistance. Genetically modified organisms, characterized by introducing foreign genetic material into an organism's genome to confer desired traits, lie outside the scope of this review. Tripathi et al. (2022) provided a comprehensive overview of the latest developments in genome editing tools, their potential applications for enhancing staple crops, and the regulatory policies governing their use in Africa. Transgenerational genome editing (TGE) refers to the ongoing use of CRISPR/Cas9 for genetic modification after a cross has occurred (Impens et al., 2022). The authors explored the concept of TGE, outlined its key applications, and provided examples of special cases to demonstrate its significance in plant genome editing research and breeding. TGE offers several applications in plant research and breeding, including editing of additional alleles in polyploid crops, creation of allelic variation, editing of target genes in recalcitrant genetic backgrounds, and combining haploid induction and gene editing. The potential of CRISPR/Cas9 technology to accelerate crop improvement by introducing desired agronomic traits has revolutionized the field of genome engineering. This technology has the advantage of expediting the delivery of improved crop varieties with desirable traits to smallholder farmers. By identifying and editing genes of interest, genome editing has been applied to various crops, enhancing their resistance to biotic and abiotic stresses, and improving product quality attributes. Genome editing has been harnessed in over 40 crops to improve agronomic traits across 25 countries (Menz et al., 2020). While genome editing holds immense promise, only a few genome edited crops such as high oleic soybean oil, non‐browning mushroom, tomato rich in gamma‐aminobutyric acid, canola, high‐amylopectin waxy corn, camelina with enhanced omega‐3 oil, and mustard green (Conscious Greens) have been approved for commercialization so far (Brown, 2023; Menz et al., 2020; Nagamine & Ezura, 2022). Notably, CGIAR centers have been making significant progress in utilizing genome editing to introduce additional traits in crops. Examples include disease (banana Xanthomonas wilt, Fusarium wilt, and banana streak virus) resistant banana; brown streak virus resistance, improved food safety (cyanide‐free), and waxy‐starch cassava; Striga resistance in sorghum [*Sorghum bicolor* (L.) Moench]; maize lethal necrosis resistant maize; disease‐resistant wheat; disease‐ and insect‐resistant and improved food safety (low arsenic and cadmium) rice; and virus‐resistant potato (Pixley et al., 2022; Tripathi et al., 2022). Incorporating genome editing technologies into current breeding programs by obliterating the obstacles associated with traditional breeding for various crops is of paramount importance to continue advancing agricultural practices and address global challenges related to food security and sustainable agriculture.

#### 5.1.3 Speed breeding

Another field of innovation is speed breeding, or rapid generation advancement (RGA). RGA can significantly speed up generation turnover and thus reduce the time it takes to develop new varieties. RGA refers to a set of techniques that involve manipulating the environmental conditions in which crop genotypes are grown (Wanga et al., 2021). Rodrmguez et al. (2023) used 150 cassava progenitors to evaluate the effectiveness of flower‐inducing technology, including photoperiod extension (Pineda, Morante, et al., 2020), pruning (Pineda, Yu, et al., 2020), and plant growth regulators (Oluwasanya et al., 2021). The authors found that time to flowering significantly decreased for all progenitors under extended lighting regimes. The most significant decrease, from 6–7 months to 3–4 months, was observed in late‐flowering progenitors. Seed production was also increased by utilizing a combination of pruning and plant growth regulators. When photoperiod extension was combined with pruning and 6‐benzyladenine (a synthetic cytokinin), a significantly higher number of fruits and seeds were produced compared to using only photoperiod extension and pruning. Edet and Ishii (2022) developed and validated an efficient speed breeding protocol for cowpea that accommodates approximately seven to eight breeding generations per year without any special equipment. They used two chambers with regulated growth conditions and cultivated new plant generations from seeds of oven‐dried immature pods. The authors found that optimal temperature, relative humidity, and light intensity improved plant growth and increased the success rate of hand pollination. Also, cultivating seeds of 11‐day‐old immature pods oven‐dried at 39°C for 2 days resulted in at least a 62% reduction between pollination and sowing of the next plant generation. Plants grown from these dried seeds showed no defects in their development. Speed breeding accelerates plant breeding and research programs (Watson et al., 2018), and GS complements this by the early identification of superior individuals. This synergy enables breeders to speed up the entire breeding process, thereby accelerating genetic gain (Chimmili et al., 2022; Jighly et al., 2019). However, speed breeding presents challenges, such as accessing suitable facilities, trained staff, and long‐term funding (Wanga et al., 2021).

### 5.2 Setting up robust data management and analytics systems

Obtaining within one genotype the target values of all quantitative traits and the entire portfolio of both quantitative and qualitative traits is challenging but represents the “true art of plant breeding.” Genotypes must be tested under varying conditions to cover the response to climate, agronomic practices, soil and water supply, and other factors that affect their performance. But this process generates vast volumes of data, and, despite its complexity, modern breeding techniques leverage increasing computational power to manage and analyze these data effectively. Today, many programs rely on standardized, digital data management to ensure consistent data integration across various dimensions, such as genomics, genealogy, phenotype, environment, and study management. Implementing digital practices such as printing barcodes for trials and nurseries, using the Field Book app (https://www.phenoapps.org/apps) for electronic data capture, developing standard operating procedures, ontologies, and controlled vocabularies, and managing inventory using seed counters and QR codes are all available tools to modernize plant breeding. These streamlined processes help enhance data accuracy, optimize resource utilization, and facilitate quicker data collection and data analysis in the breeding programs. This facilitates the use and sharing of resources and minimizes uncertainty in the selection process. Also, appropriate analytics are used to inform decision‐making. Selection based on the observed phenotypes and genotypes detected with molecular markers can be achieved by using statistical models for quantitative traits. Advances in artificial intelligence, machine learning, and robotics have the potential to transform plant breeding. Machine learning is crucial for digital agriculture, and combined with big data, it helps identify suitable genotypes for specific environments (Kuriakose et al., 2020). As the amount of data generated by breeding programs in experiments continues to grow, new and up‐to‐date tools and techniques for managing, sharing, analyzing, visualizing, and interpreting these vast datasets (phenotypic, molecular, spatial, soil, weather, etc.) are needed. Overall, implementing breeding database systems, utilizing cloud‐based platforms, and employing advanced data analytics can streamline data management and accelerate data‐driven decision‐making processes. CGIAR has adopted this approach to develop the Enterprise Breeding System (EBS), an open‐source and cloud‐based breeding informatics system. EBS is a tool for crop breeding programs in Africa, Asia, and Latin America. It helps digitize breeding programs, manage data in real‐time, enable secure data‐sharing, and perform advanced analytics for informed breeding decisions and faster breeding cycles. Similar platforms such as Breedbase (Morales et al., 2022), the Breeding Management System of the Integrated Breeding Platform (https://bmspro.io/), and other commercial products also exist, offering alternative solutions for managing breeding data and facilitating informed breeding decisions and accelerated breeding cycles.

### 5.3 Including producer and consumer insights into the varietal selection processes

Developing effective varietal selection processes in plant breeding requires a strong focus on customer insights to ensure that new varieties meet specific needs and preferences. To achieve this, breeders should engage customers and other stakeholders through surveys, interviews, and focus groups to gather valuable feedback on desired traits, market demands, and cultivation practices. Notably, recent studies (Naa Ashiokai Prempeh et al., 2024; Nakitto et al., 2023; Nchanji et al., 2023; Regassa et al., 2023) exemplify the practical application of customer data in this context. Guided by the TPP framework, breeders can identify target traits and establish performance benchmarks that harmonize with market demands and cultivation practices. They can integrate customer data with phenotypic and genotypic information to design breeding strategies to develop varieties that meet the specified criteria outlined in the TPP. Continuous stakeholder feedback and on‐farm trials are then instrumental in evaluating candidate varieties’ real‐world performance throughout the breeding process. This iterative approach allows breeders to continuously assess customer satisfaction, track market acceptance, and make improvements to meet evolving demands. This can be facilitated using the triadic comparison of technology (Tricot) approach (van Etten et al., 2020) in harnessing farm data to inform better breeding decisions and enhance marketing outcomes. The approach involves each farmer assessing three unidentified varieties selected from a broader range tested within the community. Farmers are asked to rank the three varieties according to their preferences, considering factors like yield and quality. One advantage of the Tricot method is its ability to engage a larger number of farmers, leading to testing in various environments, given that not all farmers evaluate identical sets of varieties (Moyo et al., 2021). However, one challenge of this approach is the difficulty in obtaining quantitative data instead of rankings provided by farmers. To address this limitation, various methods are being tested to collect reliable and valid farmer‐led yield estimations for crops such as maize, common bean (*Phaseolus vulgaris* L.), and cassava (de Sousa et al., 2024). Nevertheless, the insights gained from on‐farm trials not only facilitate product profile enhancements but also inform investment decisions and instill confidence among seed companies regarding the productivity, market potential, and geographic suitability of preferred varieties. Including tricot in breeding programs has helped develop and validate on‐farm trial protocols based on TPPs. These protocols include core traits, socioeconomic indicators, and plot characterization, which facilitate the scaling and pooling of data across trials, geographies, and crops (de Sousa et al., 2024).

### 5.4 Tracking operational costs

Maximizing the genetic gain rate over successive generations, as outlined in the breeder's equation, underscores the importance of effective cost management in plant breeding programs (Cobb et al., 2019). Operational costs for plant breeding programs can vary significantly depending on crop species, infrastructure, methods, and objectives, presenting breeders with an optimization challenge. To address this, breeders must balance genetic gain per dollar invested and the rate of genetic gain, which can fluctuate due to changes in input prices, program scale, breeding goals, and technological advances. Tracking operational costs is a crucial first step in this optimization problem. By employing cost accounting systems like activity‐based costing, breeders can monitor expenses related to personnel, materials, equipment, services, and facilities. Rigorous budget monitoring and control mechanisms ensure that breeding programs operate within their financial constraints, enhancing efficiency and resource allocation. Furthermore, conducting cost‐benefit analyses allows breeders to assess the economic viability of different breeding strategies and investments, ensuring that resources are allocated to initiatives with the highest potential for enhancing genetic gain. Utilizing online tools, such as the UQ Breeding Program Costing Tool (The University of Queensland, 2020), facilitates the estimation of breeding program costs. The costing tool software has a range of uses, such as identifying areas with high costs within breeding programs. It can also conduct “What If?” scenarios to evaluate the cost implications of different strategies, accurately cost fee‐for‐service activities such as pathology screening or trials, develop comprehensive breeding program budgets, create business cases for capital item procurement, and allow the sharing of cost databases with management for informed decision‐making. Additionally, benchmarking against industry standards and best practices provides further insights into program performance and identifies areas for improvement. Tracking the operational costs enables breeders to pinpoint cost drivers and prioritize activities that contribute most to breeding progress.

### 5.5 Establishing strong collaboration and capacity development

Capacity building is essential for the continuous advancement of plant breeding programs. To effectively enhance capacity, initiatives must prioritize comprehensive training and educational opportunities to breeders, technicians, and support staff with essential skills and knowledge. This includes training in modern breeding techniques, sound experimental designs, proficient data management, and appropriate analytics, genomics, and breeding operations. Opportunities to revisit initiatives such as the Integrated Breeding Multiyear Course, previously conducted by the Integrated Breeding Platform, hold promise for advancing breeding capabilities. This comprehensive 3‐year program equipped breeders from developing countries with essential skills in molecular breeding techniques, data management, and analytics. Structured with both online support and annual face‐to‐face training sessions tailored to specific regions—Eastern and Southern Africa, West and Central Africa, and South and Southeast Asia—the course provided a robust platform for practical learning. Participants engaged directly in breeding activities, applying newfound knowledge, and gaining proficiency in emerging tools and methodologies.

For the next generation of plant breeders in SSA, institutions such as the African Centre for Crop Improvement (https://acci.org.za/) and the West African Centre for Crop Improvement (https://wacci.ug.edu.gh/) aspire to provide extensive training at scale for African breeders focused on African crops within the continent. These initiatives collaborate closely with advanced universities like Cornell and receive support from the Rockefeller Foundation, the Alliance for a Green Revolution in Africa, and the Bill & Melinda Gates Foundation (BMGF), among others, offering significant comparative advantages.

Collaboration is equally important and catalyzes driving progress and innovation. Plant breeding must form interactive communities of practice to consolidate demand and stimulate the development of low‐cost techniques and services (Cobb et al., 2019). By forging partnerships among different breeding programs and institutions, valuable resources and expertise can be shared, leading to accelerated advancements in breeding efforts. An example is the CGIAR‐NARES alignment, which has significantly contributed to boosting genetic gains, mainly through germplasm exchange, harmonization of research agendas, and adoption of emerging technologies. As for an example, the Africa Rice Center (AfricaRice) uses task forces to research with NARES and develop new rice technologies across Africa. The task forces focus on six areas: breeding, agronomy, postharvest, policy, gender, and mechanization. They work together to pool scarce resources and foster national involvement (https://www.africarice.org/africa‐wide‐rice‐task‐forces). Another example is the effective collaboration between germplasm banks and end‐users, which is crucial for successfully utilizing plant genetic resources. The CGIAR Genebanks (https://www.cgiar.org/initiative/genebanks/) aim to conserve and provide access to plant genetic resources for climate‐adapted crops and resilient food systems. Similarly, the National Plant Germplasm System of the United States Department of Agriculture (Byrne et al., 2018) provides improved cultivars, breeding materials, landraces, and crop wild relatives. These genetic resources, accompanied by passport and evaluation data accessible to plant breeders, are the outcome of robust international collaboration efforts.

Additionally, public–private partnerships are crucial in leveraging global expertise, accessing funding opportunities, and accelerating the development and dissemination of improved plant varieties. Bayer has been collaborating with International Institute of Tropical Agriculture through a BMGF‐funded project to modernize breeding programs and operations, particularly in designing, developing, and disseminating demand‐driven improved products.

## 6 CONCLUSION

Modern techniques such as GS, gene editing, and speed breeding are essential to ensure that plant breeding programs contribute to building resilient, nutrition‐focused, and productive crop‐based food systems. As a general prerequisite, handling the vast and varied datasets required for these initiatives builds on robust data management and analytics systems around digital platforms and the LMM. Including customer insights in the varietal selection process will ensure that new varieties meet specific market needs and demands, enhancing adoption rates and successful marketing of new varieties. Collaborations and capacity building are critical, with international collaborations and public–private partnerships offering avenues for expertise exchange, resource sharing, and access to funding opportunities. Monitoring the success and progress of breeding programs requires continued, annual measurement of realized genetic gain. This is a process that requires meticulous data collection and analysis. By harnessing historical data from routine MET and utilizing advanced statistical methods, breeders can differentiate genetic from nongenetic trends, thereby estimating the true impact of their breeding efforts.

## AUTHOR CONTRIBUTIONS

**Ibnou Dieng**: Conceptualization; data curation; formal analysis; investigation; methodology; software; validation; writing—original draft; writing—review and editing. **Brian Gardunia**: Conceptualization; investigation; methodology; software; validation; writing—original draft; writing—review and editing. **Giovanny Covarrubias‐Pazaran**: Investigation; methodology; software; writing—original draft; writing—review and editing. **Dorcus C. Gemenet**: Investigation; methodology; writing—original draft; writing—review and editing. **Bodo Trognitz**: Investigation; methodology; writing—original draft; writing—review and editing. **Sam Ofodile**: Investigation; methodology; software; writing—original draft; writing—review and editing. **Kayode Fowobaje**: Investigation; methodology; software; writing—original draft; writing—review and editing. **Solomon Ntukidem**: Investigation; methodology; software; writing—original draft; writing—review and editing. **Trushar Shah**: Investigation; methodology; writing—original draft; writing—review and editing. **Simon Imoro**: Investigation; methodology; writing—original draft; writing—review and editing. **Leena Tripathi**: Investigation; methodology; validation; writing—original draft; writing—review and editing. **Hapson Mushoriwa**: Investigation; methodology; validation; writing—original draft; writing—review and editing. **Ruth Mbabazi**: Investigation; methodology; writing—original draft; writing—review and editing. **Stella Salvo**: Investigation; methodology; validation; writing—original draft; writing—review and editing. **John Derera**: Investigation; methodology; validation; writing—original draft; writing—review and editing.

## CONFLICT OF INTEREST STATEMENT

The authors declare no conflicts of interest.

## Data Availability

The scripts generated and the data analyzed for this study are available in the GitHub repository: https://github.com/Biometrics‐IITA/Estimating‐Realized‐Genetic‐Gain and in the Dryad repository: https://doi.org/10.5061/dryad.k98sf7mfr.
